# Supplementary figures and images for: Widespread phages of endosymbionts: Phage WO genomics and the proposed taxonomic classification of Symbioviridae
Source: PLoS Genet. 2022 Jun 6;18(6):e1010227. doi: 10.1371/journal.pgen.1010227 (PMC9203015; doi:10.1371/journal.pgen.1010227)

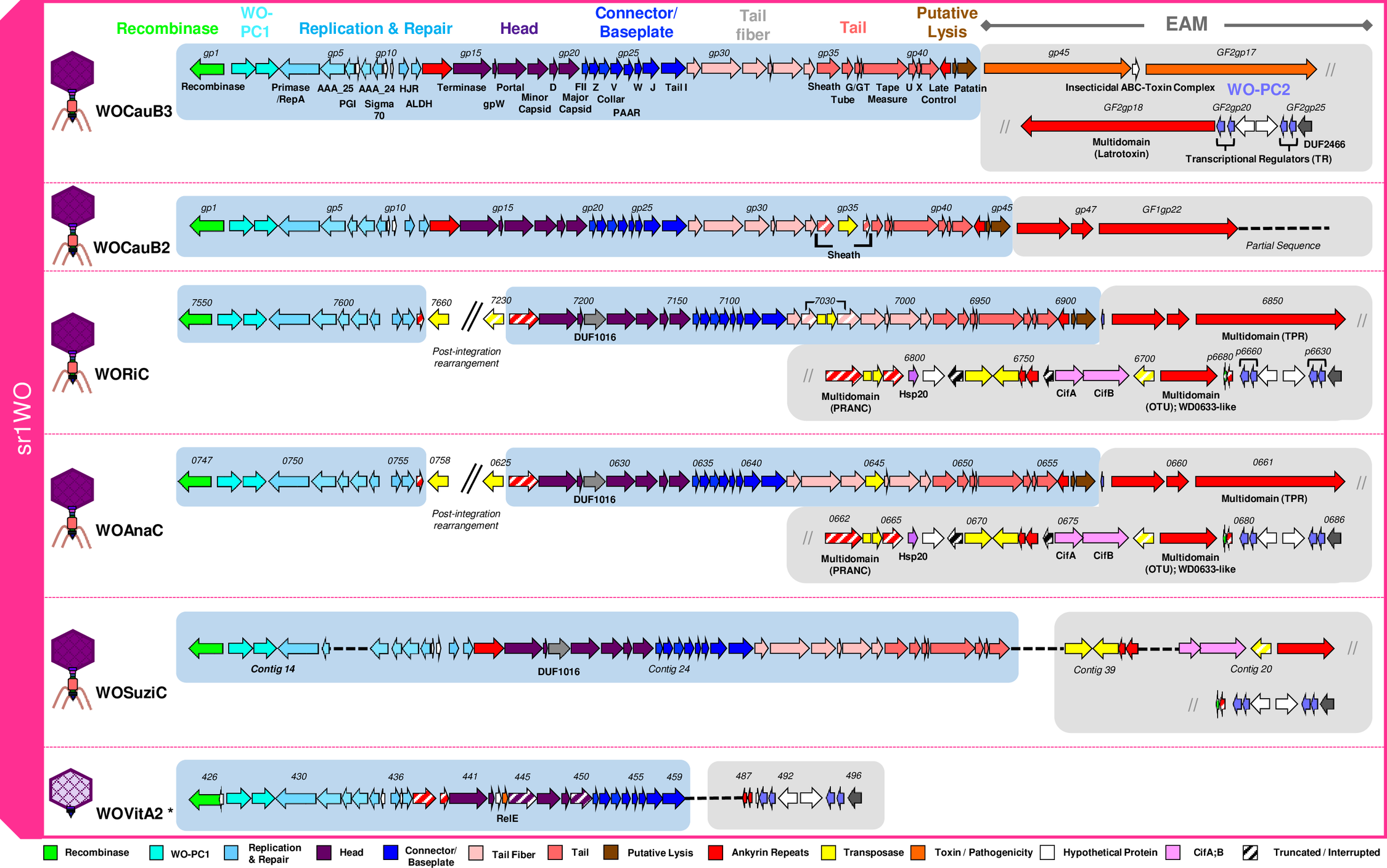

Supplement: S1 Fig — Genome maps of sr1WO prophage regions where genes are drawn to scale in forward and reverse directions. Predicted physical structures are illustrated to the left of each genome. All genomes contain tail modules with the exception of the partial WOVitA2 sequence. Prophage WO Core Genes are shaded in blue and predicted EAM genes are shaded in gray. Genes of similar function are similarly color-coded according to the figure legend. Locus tags, if available, are listed in italics above the genes. The large, black diagonal lines between the recombinase and transposase in WORiC and WOSuziC represent post-integration rearrangement of the prophage region in the Wolbachia chromosome. Dashed lines represent breaks in the assembly whereas small diagonal lines represent a continuation of the genome onto the next line. Arrows with diagonal stripes represent genes that may be pseudogenized relative to homologs in other prophage WO genomes. The putative function for each structural gene is discussed in S1 Text. * Indicates a partial sequence and/or highly degraded genome that may be considered a WO-like Island; gene content, module synteny, and recombinase typing support a putative sr1WO-origin. (TIF) [file pgen.1010227.s001.tif]

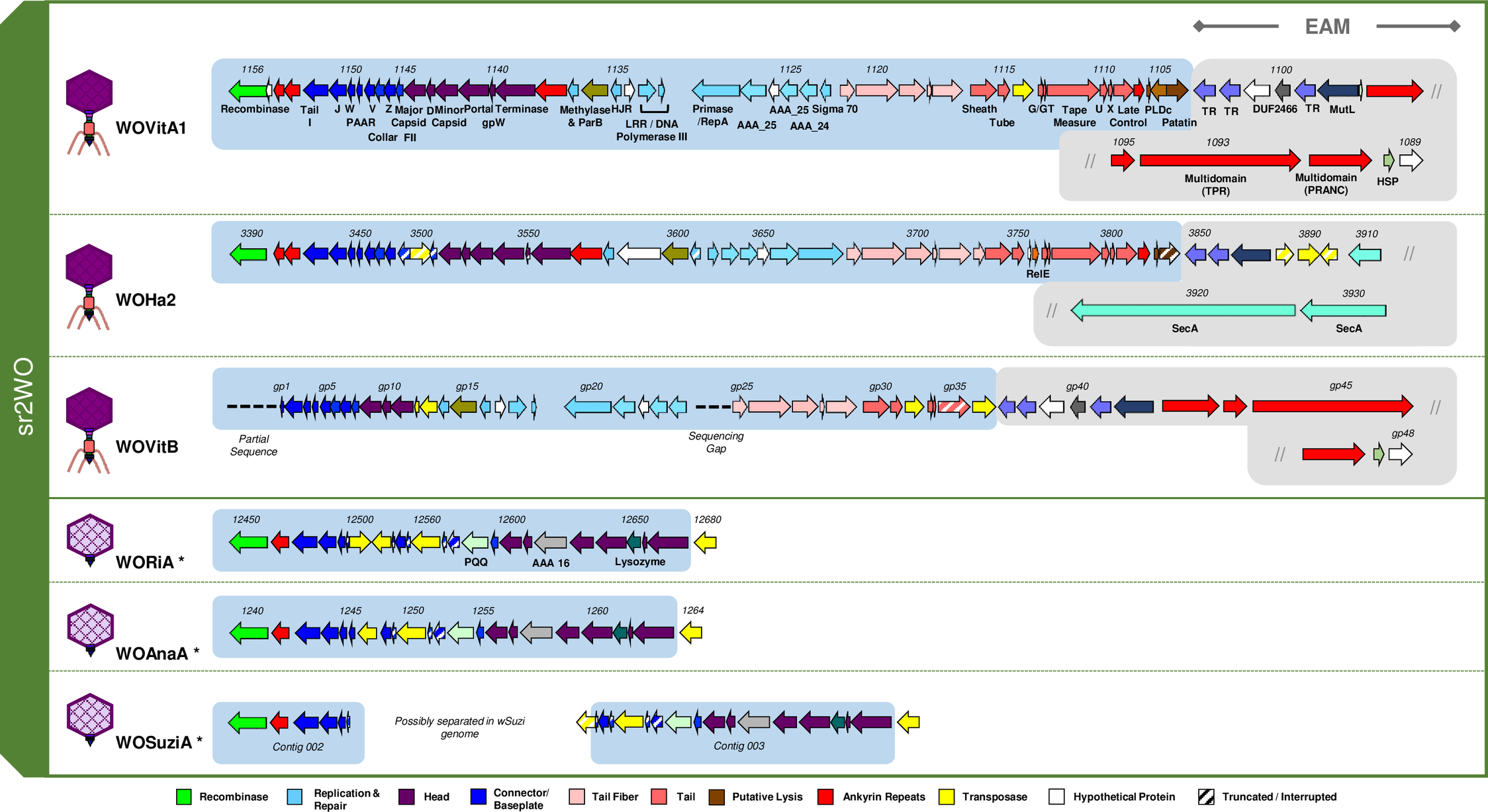

Supplement: S2 Fig — Genome maps of sr2WO prophage regions where genes are drawn to scale in forward and reverse directions. Predicted physical structures are illustrated to the left of each genome. WOVitA1-like prophage genomes encode all structural modules (shaded in blue) and an EAM (shaded in gray) whereas WORiA-like prophage genomes encode an intact head module, recombinase, lysozyme, AAA16, and disrupted connector. They lack most other modules. Genes of similar function are similarly color-coded according to the figure legend. Locus tags, if available, are listed in italics above the genes. Dashed lines represent breaks in the assembly whereas small diagonal lines represent a continuation of the genome onto the next line. Arrows with diagonal stripes represent genes that may be pseudogenized relative to homologs in other prophage WO genomes. The putative function for each structural gene is discussed in S1 Text. * Indicates a partial sequence or highly degraded genome that may be considered a WO-like Island; gene content, module synteny, and recombinase typing support a putative sr2WO-origin. (TIF) [file pgen.1010227.s002.tif]

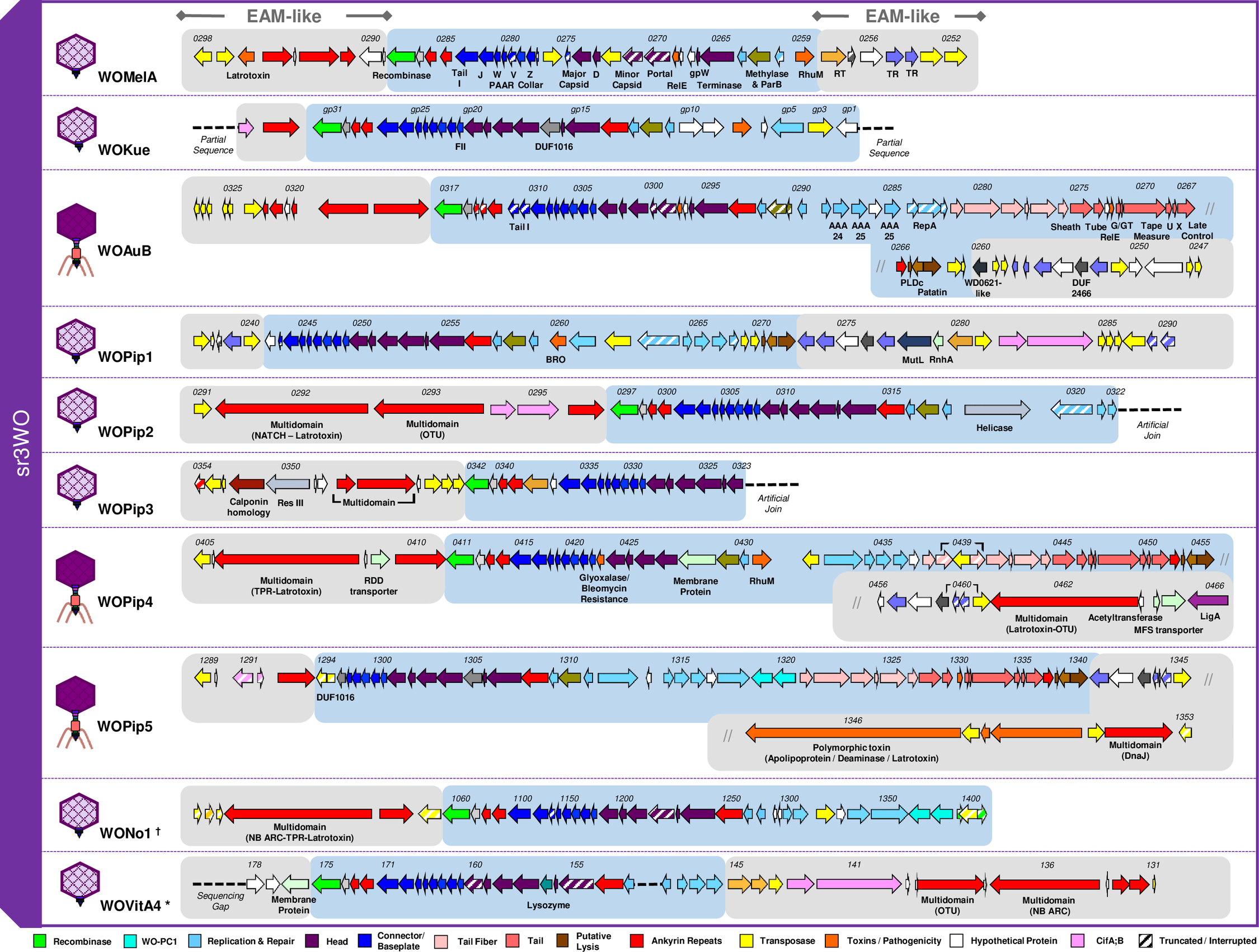

Supplement: S3 Fig — Genome maps of sr3WO prophage regions where genes are drawn to scale in forward and reverse directions. Three wPip prophages exist as one contiguous prophage region in the Wolbachia genome and are illustrated here as WOPip1, WOPip2, and WOPip3 (based on [110]). Predicted physical structures are illustrated to the left of each genome. Prophage WO Core Genes are shaded in blue and predicted EAM genes are shaded in gray. Genes of similar function are similarly color-coded according to the figure legend. sr3WO is comprised of highly variable genomes that are often flanked by mobile elements (transposases are shown in yellow). They generally contain a recombinase, connector/baseplate, head, and EAM with only a few genomes encoding a complete tail. Prophages in this group often contain cifA;B (pink). Locus tags are listed in italics above the genes. Dashed lines represent breaks in the assembly whereas small diagonal lines represent a continuation of the genome onto the next line. Arrows with diagonal stripes represent genes that may be pseudogenized relative to homologs in other prophage WO genomes. The putative function for each structural gene is discussed in S1 Text. * Indicates a partial sequence or highly degraded genome that may be considered a WO-like Island; gene content, module synteny, and recombinase typing support a putative sr3WO-origin. † The WONo1 region is a chimera between a 5’-sr3WO and 3’-sr1WO. Definitive boundaries are unknown. (TIF) [file pgen.1010227.s003.tif]

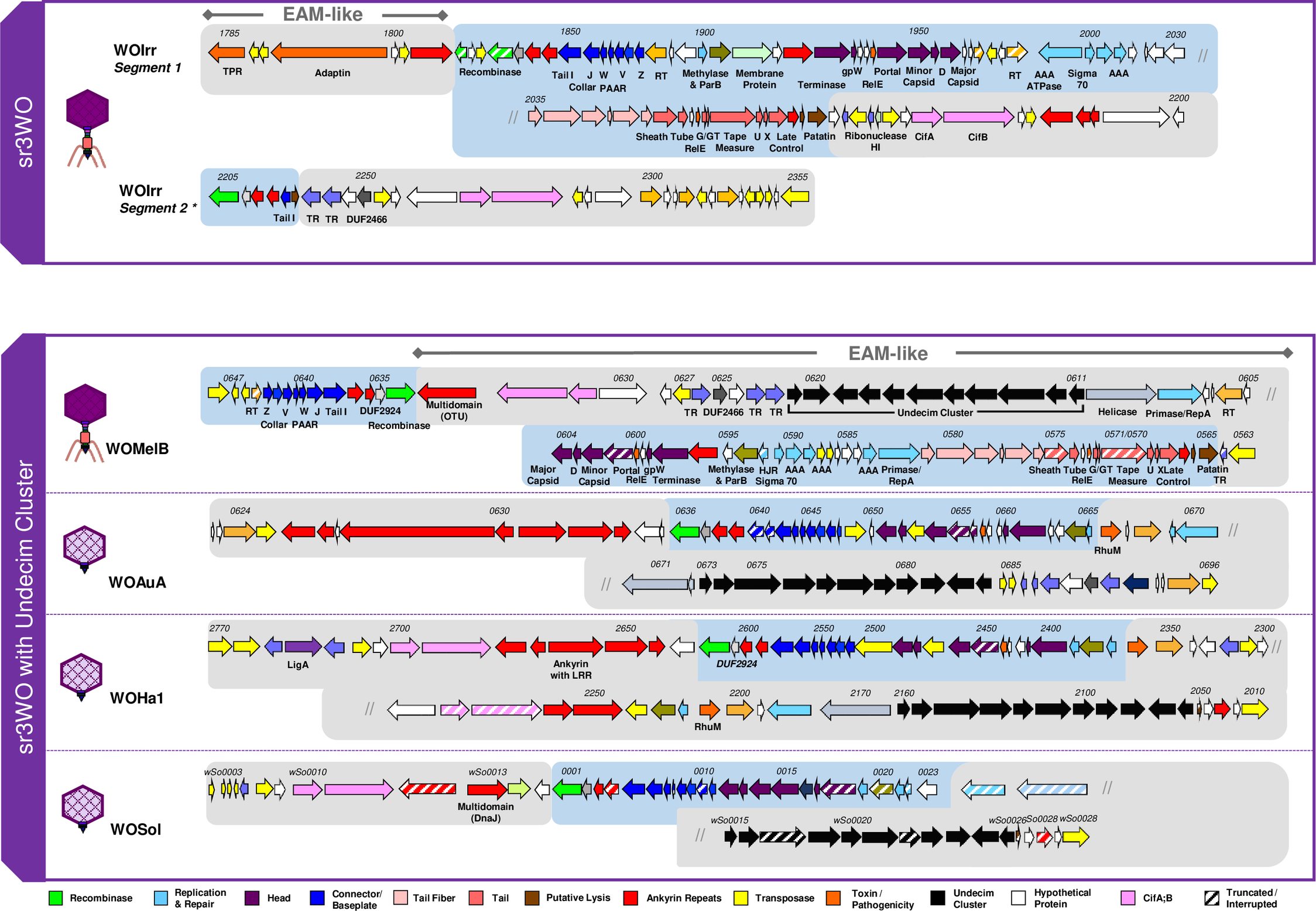

Supplement: S4 Fig — Genome maps of sr3WO prophage regions where genes are drawn to scale in forward and reverse directions. WOIrr is one contiguous prophage region in the Wolbachia genome that is illustrated here as Segment 1 and Segment 2. A subset of sr3WO prophages is further categorized by the presence of a highly conserved WD0611-WD0621 like region, termed the Undecim Cluster (black). Predicted physical structures are illustrated to the left of each genome. Prophage WO Core Genes are shaded in blue and predicted EAM genes are shaded in gray. Genes of similar function are similarly color-coded according to the figure legend. sr3WO is comprised of highly variable genomes that are often flanked by mobile elements (transposases are shown in yellow). Prophages in this group often contain cifA;B (pink). Locus tags are listed in italics above the genes. Dashed lines represent breaks in the assembly whereas small diagonal lines represent a continuation of the genome onto the next line. Arrows with diagonal stripes represent genes that may be pseudogenized relative to homologs in other prophage WO genomes. The putative function for each structural gene is discussed in S1 Text. * Indicates a partial sequence or highly degraded genome that may be considered a WO-like Island; gene content, module synteny, and recombinase typing support a putative sr3WO-origin. (TIF) [file pgen.1010227.s004.tif]

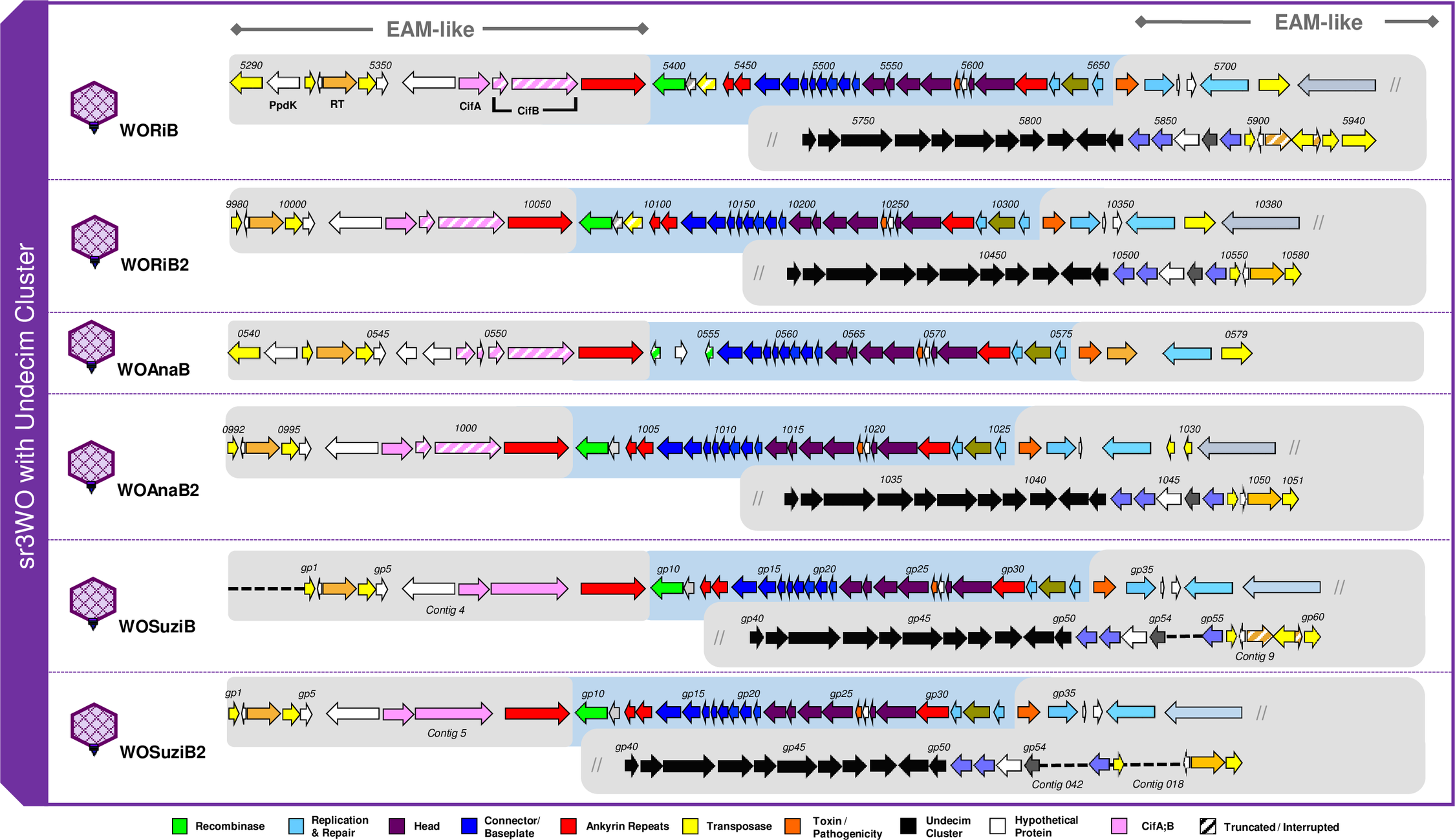

Supplement: S5 Fig — Genome maps of sr3WO prophage regions where genes are drawn to scale in forward and reverse directions. This subset of sr3WO prophages is further categorized by the presence of a highly conserved WD0611-WD0621 like region, termed the Undecim Cluster (black). Predicted physical structures are illustrated to the left of each genome. Prophage WO Core Genes are shaded in blue and predicted EAM genes are shaded in gray. Genes of similar function are similarly color-coded according to the figure legend. sr3WO is comprised of highly variable genomes that are often flanked by mobile elements (transposases are shown in yellow). Prophages in this group often contain cifA;B (pink). Locus tags are listed in italics above the genes. Dashed lines represent breaks in the assembly whereas small diagonal lines represent a continuation of the genome onto the next line. Arrows with diagonal stripes represent genes that may be pseudogenized relative to homologs in other prophage WO genomes. The putative function for each structural gene is discussed in S1 Text. (TIF) [file pgen.1010227.s005.tif]

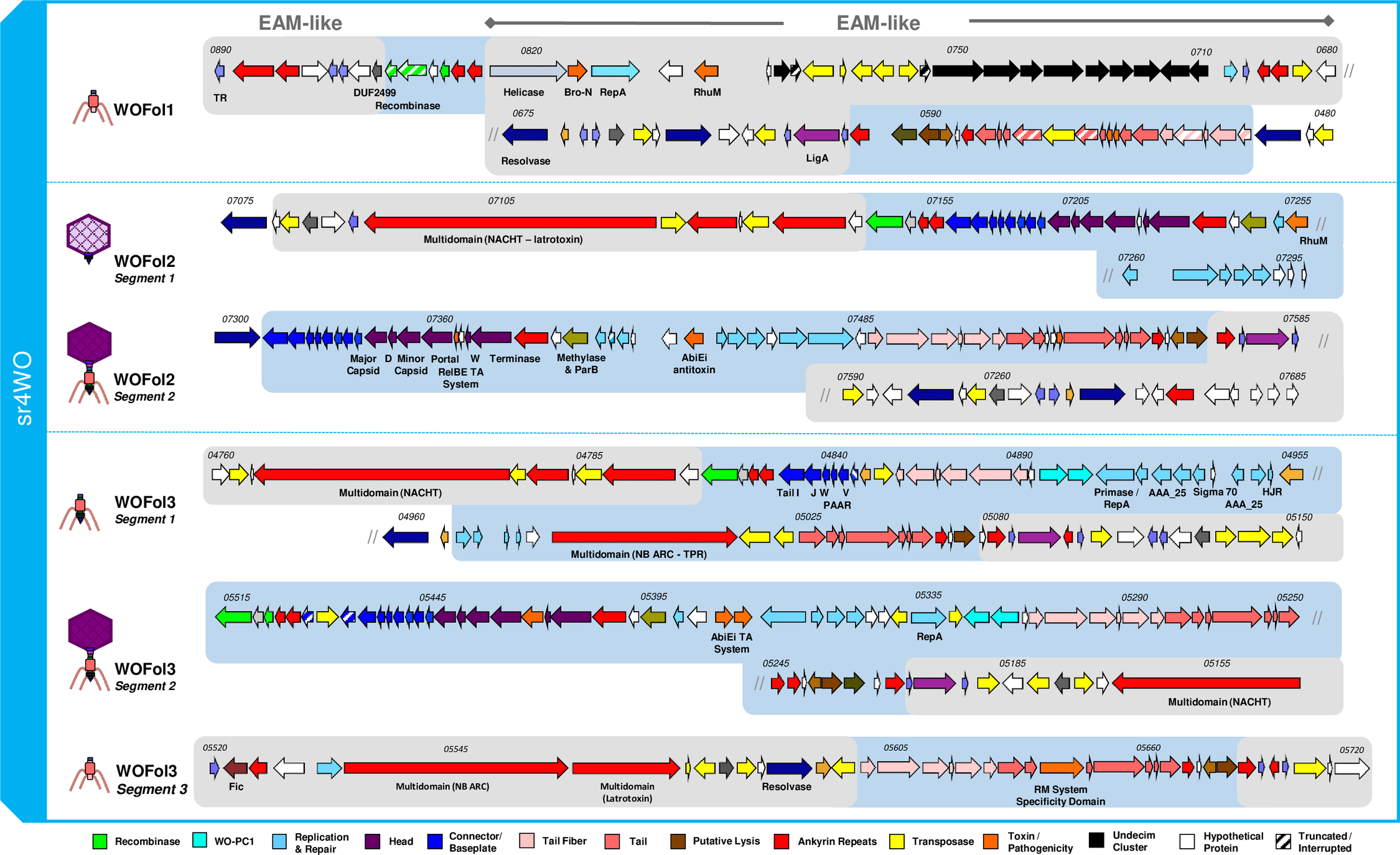

Supplement: S6 Fig — Genome maps of sr4WO prophage regions where genes are drawn to scale in forward and reverse directions. To date, sr4WO prophages have only been identified in the parthenogenic strain of Folsomia candida, wFol. WOFol2 is one contiguous prophage region in the Wolbachia genome that is illustrated here as Segment 1 and Segment 2. Likewise, the WOFol3 prophage region is illustrated as three segments. Predicted physical structures are illustrated to the left of each genome. Prophage WO Core Genes are shaded in blue and predicted EAM genes are shaded in gray. Genes of similar function are similarly color-coded according to the figure legend. Locus tags are listed in italics above the genes. Small diagonal lines represent a continuation of the genome onto the next line. Arrows with diagonal stripes represent genes that may be pseudogenized relative to homologs in other prophage WO genomes. The putative function for each structural gene is discussed in S1 Text. (TIF) [file pgen.1010227.s006.tif]

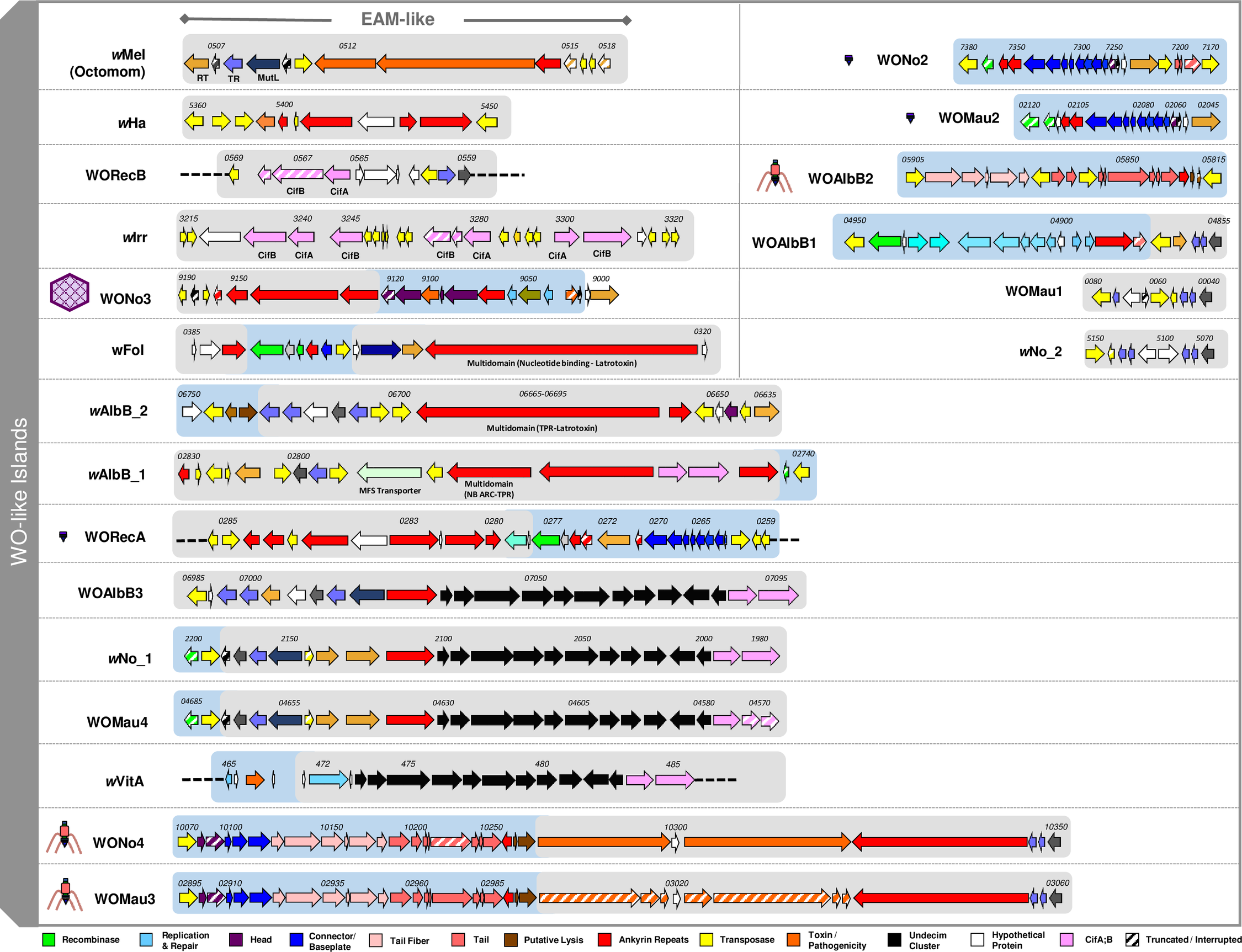

Supplement: S7 Fig — Genome maps of WO-like Islands where genes are drawn to scale in forward and reverse directions. These regions contain only one structural module and/or group of WO-related genes. Regions flanked by assembly breaks (i.e., WORecB, WORecA, and wVitA) are tentatively classified as WO-like Islands due to lack of a full-length prophage in the genome assembly. Names are based on the original author’s description. If it was identified as a prophage in the genome announcement, the reported WO name is listed here. Otherwise, the name simply refers to the encoding Wolbachia genome. Many WO-like Islands contain cifA;B; some Islands (i.e., wNo, wVitA, WOMau4, and WOAlbB3) contain both Type III cifA;B (pink) and the Undecim Cluster (black). Predicted physical structures are illustrated to the left of each genome. Prophage WO Core Genes are shaded in blue and predicted EAM genes are shaded in gray. Genes of similar function are similarly color-coded according to the figure legend. Locus tags are listed in italics above the genes. Dashed lines represent breaks in the assembly. Arrows with diagonal stripes represent genes that may be pseudogenized relative to homologs in other prophage WO genomes. The putative function for each structural gene is discussed in S1 Text. (TIF) [file pgen.1010227.s007.tif]

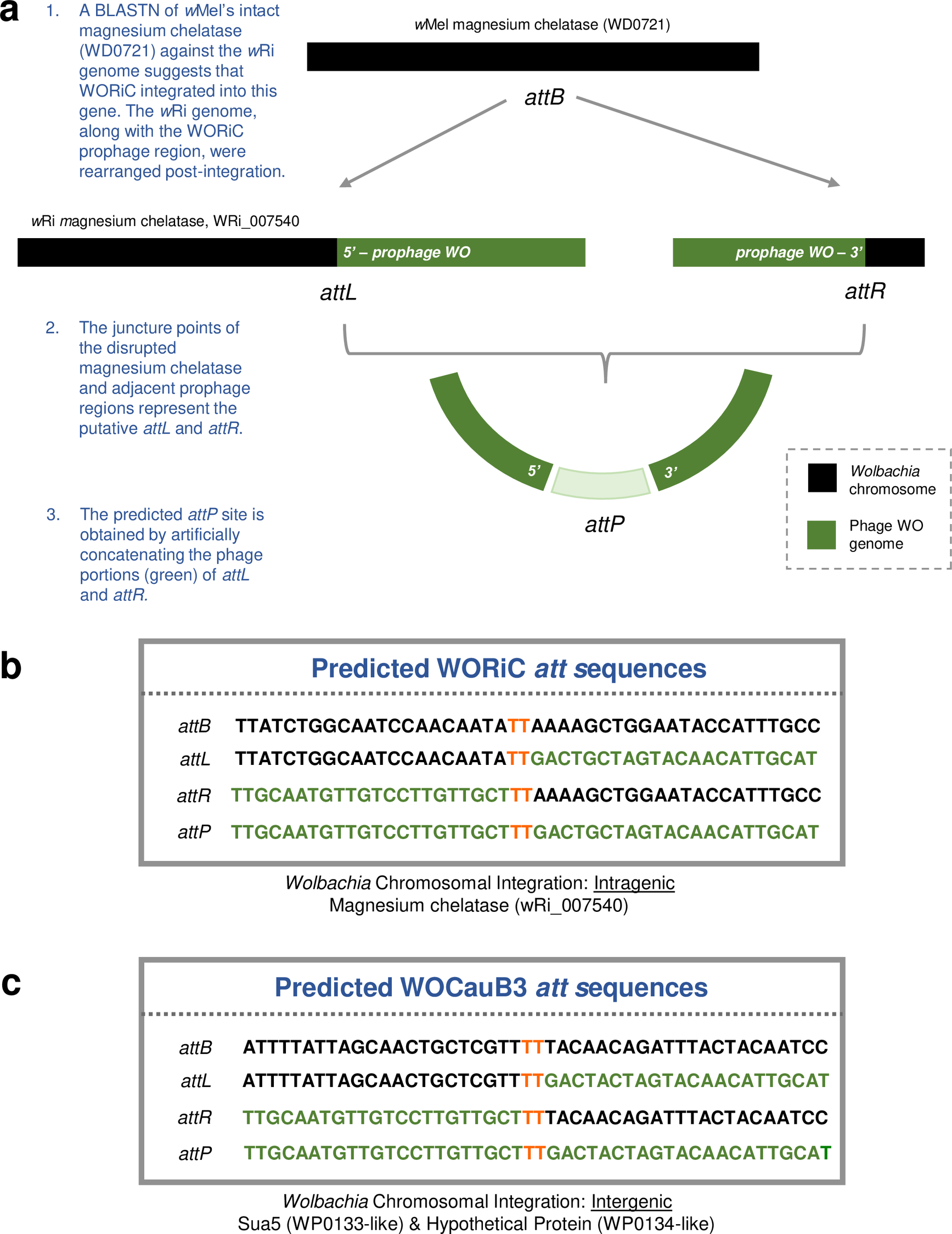

Supplement: S8 Fig — An integrated prophage sequence contains left and right attachment sites (attL and attR, respectively) at the points of chromosomal integration. Half of the att site is phage-derived (green); the other half is bacterial derived (black). If the DNA sequence of the bacterial attachment site (attB, black) is known, a nucleotide alignment of the intact sequence with the integrated prophage genome will correlate with 5’- (attL) and 3’- (attR) prophage boundaries. (a) WORiC, a member of sr1WO, integrates into wRi’s magnesium chelatase gene. By aligning an intact copy of this gene (WD0721) from closely related wMel that does not harbor sr1WO, (b) the juncture points of the disrupted magnesium chelatase indicate the attL and attR sites for the WORiC prophage region within the wRi genome. (b) The phage attachment site (attP, green) is predicted in silico by concatenating the non-Wolbachia portions of the attL and attR sites. (c) Likewise, this method can also be applied when the bacterial integration locus is intergenic. The homologous intergenic region of closely related, sr1WO-free wPip can be used to predict att sites for WOCauB3. Nucleotides in orange represent a common region, O, that is shared by all four att sites. This method was adapted from [39] where the attP site was used to predict the attB site of WOVitA1. (TIF) [file pgen.1010227.s008.tif]

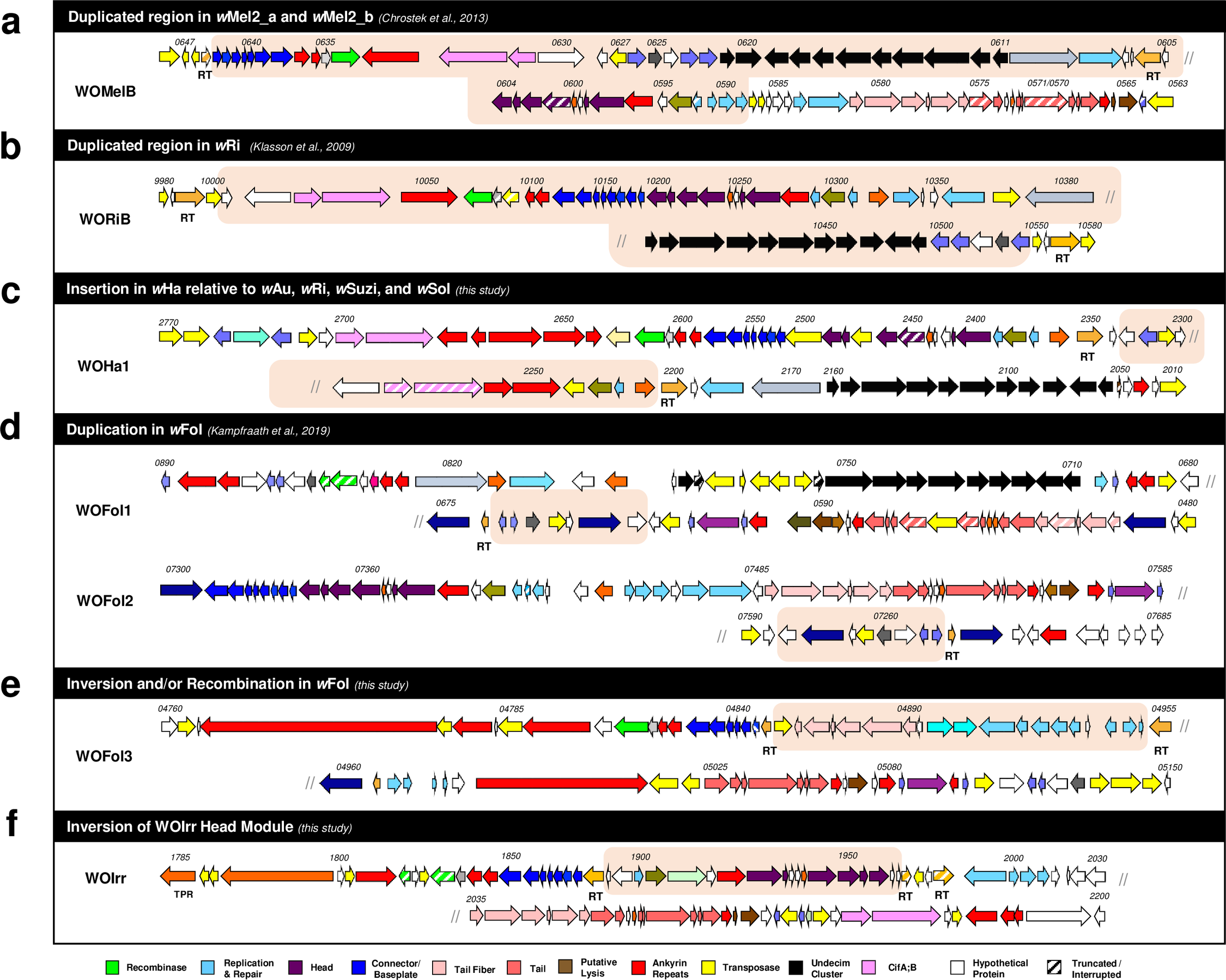

Supplement: S9 Fig — (a) The WOMelB prophage genomes of wMel2_a and wMel2_b are duplicated relative to the wMel reference genome [72]. (b) The entire WORiB prophage region is duplicated in wRi [19]. (c) WOHa1 encodes a second, pseudogenized cifA;B-containing region relative to closely related WOAuA, WORiB, WOSuziB, and WOSol prophages. (d) A ligase-containing region is duplicated in wFol’s WOFol1 and WOFol2 [56]. (e) Based on homology to other prophage regions (Fig 2), the connector/baseplate should be adjacent to a head module and the WOPC-2 and replication genes should be oriented in the opposite direction; this indicates a likely insertion and/or recombination in the WOFol3 prophage region. (f) The WOIrr head module is inverted relative to other sr3WOs. Genes are illustrated as arrows; putative gene annotations are labeled in S1–S7 Figs. In each example, the regions of chromosomal rearrangement are highlighted in light orange and flanked by at least one RT. (TIF) [file pgen.1010227.s009.tif]

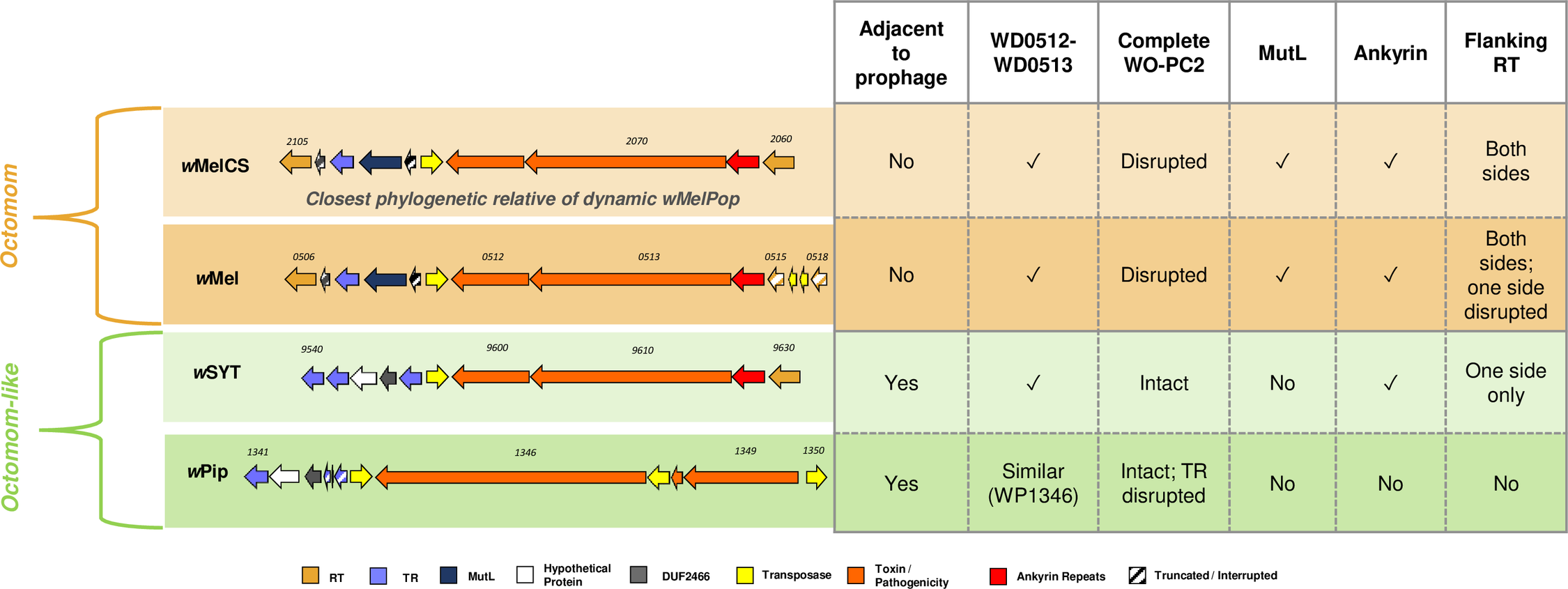

Supplement: S10 Fig — Octomom (orange) and Octomom-like (green) regions are illustrated for wMelCS, wMel, wSYT clade, and wPip. Characteristics of each region are listed next to the genome schematic. Notably, the wMelCS genome, representative of the dynamic wMelPop, is distinguished from other variants by intact, flanking reverse transcriptases of group II intron origin (RT) on both sides. wPip, the only Wolbachia Supergroup B variant, is the most divergent and not associated with an RT, MutL or ankyrin repeat. Rather it is adjacent to WP1349, another gene that has been horizontally transferred between phage and arthropod [71]. (TIF) [file pgen.1010227.s010.tif]

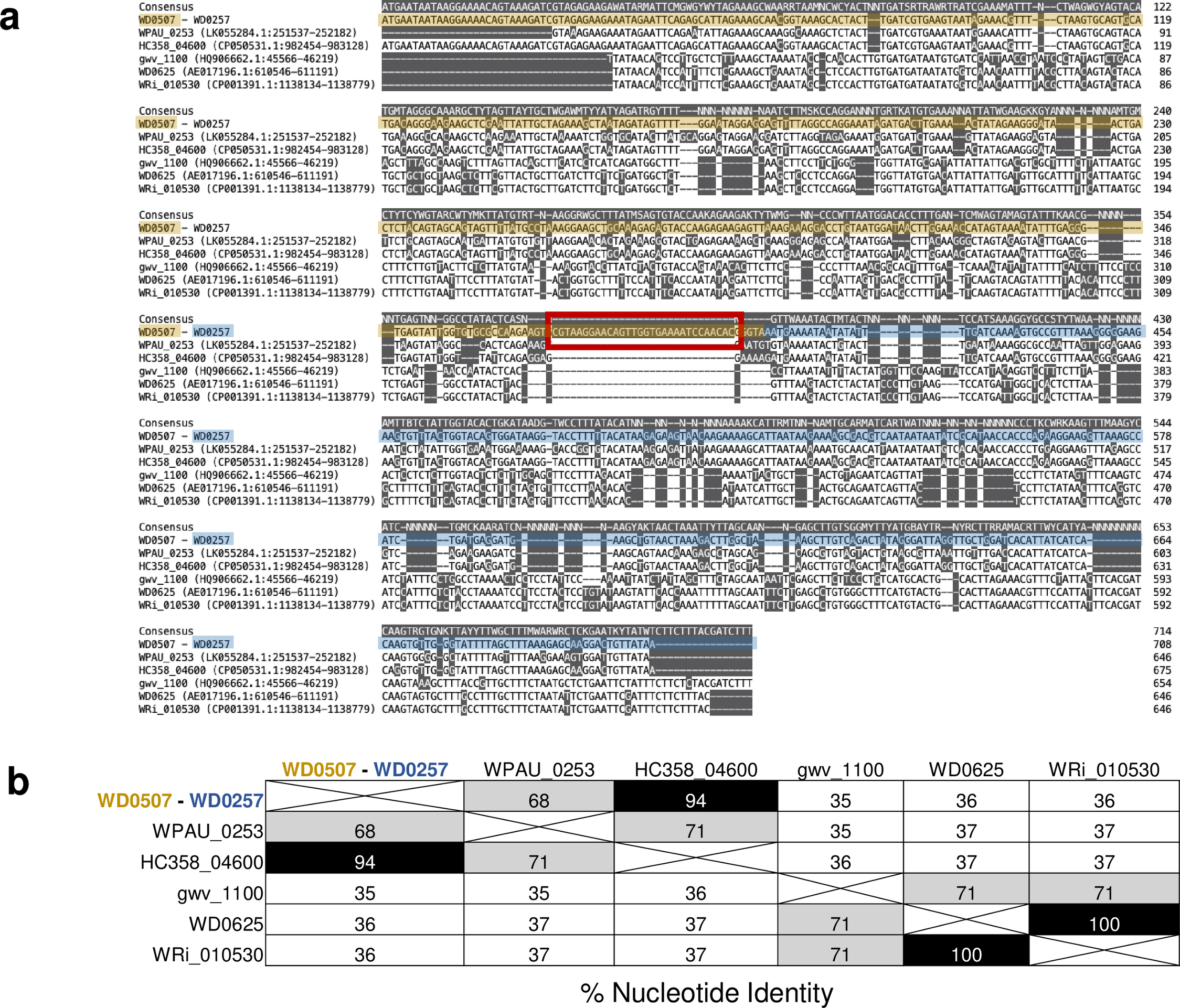

Supplement: S11 Fig — (a) A nucleotide alignment of concatenated WD0507 (Octomom) and WD0257 (WOMelA) illustrates homology with intact DUF2466 genes of similar WO-PC2 modules, except for a 30-bp insertion at the 3’-end of WD0507 (highlighted in red). WD0507 is shaded in gold; WD0257 is shaded in blue. Disagreements relative to consensus (excluding ambiguous disagreements) are shaded in gray. (b) A distance matrix of the alignment confirms that the putative ancestral DUF2466 shares 94% and 68% nucleotide identity with homologous WO-PC2 modules in wStv (HC358_04600) and wAu (WPAU_0253), respectively. (TIF) [file pgen.1010227.s011.tif]

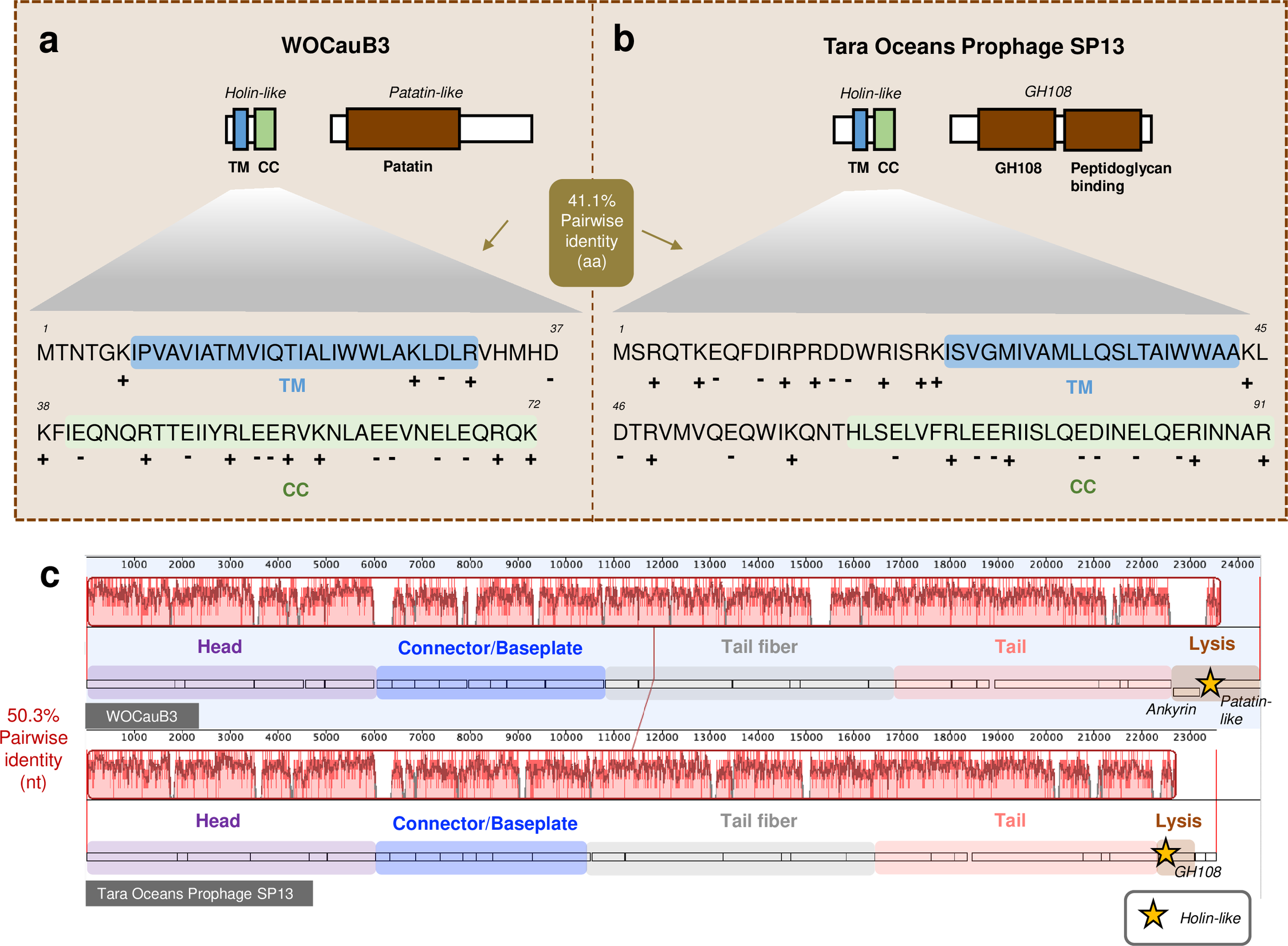

Supplement: S12 Fig — Adjacent to the tail module of most prophage WO variants are three phage lysis candidates: ankyrin repeat containing protein (not shown), holin-like, and patatin-like phospholipase. (a) Similar to canonical holins, the prophage WO gene product encodes a single N-terminal transmembrane domain with no predicted charge. It is smaller than 150 amino acid residues, features a C-terminal coiled coil motif, and has a highly charged C-terminal domain. Unlike canonical holins, however, it is adjacent to a patatin-like gene rather than a characterized endolysin. (b) The prophage WO holin-like peptide shares 41.1% amino acid identity to a homolog in the non-Wolbachia prophage from the Tara Oceans Project that is directly adjacent to a GH108 lysozyme (complete genome illustrated in Fig 6). (c) A Mauve alignment of these genomic regions (core phage modules only; EAM not included) indicates 50.3% nucleotide identity across the majority of the sequence, including the holin-like gene (marked with a gold star). The similarity of these prophages suggest that prophage WO may utilize a similar holin-like gene with a different lytic enzyme (i.e., patatin rather than lysozyme) to lyse the bacterial cell. (TIF) [file pgen.1010227.s012.tif]

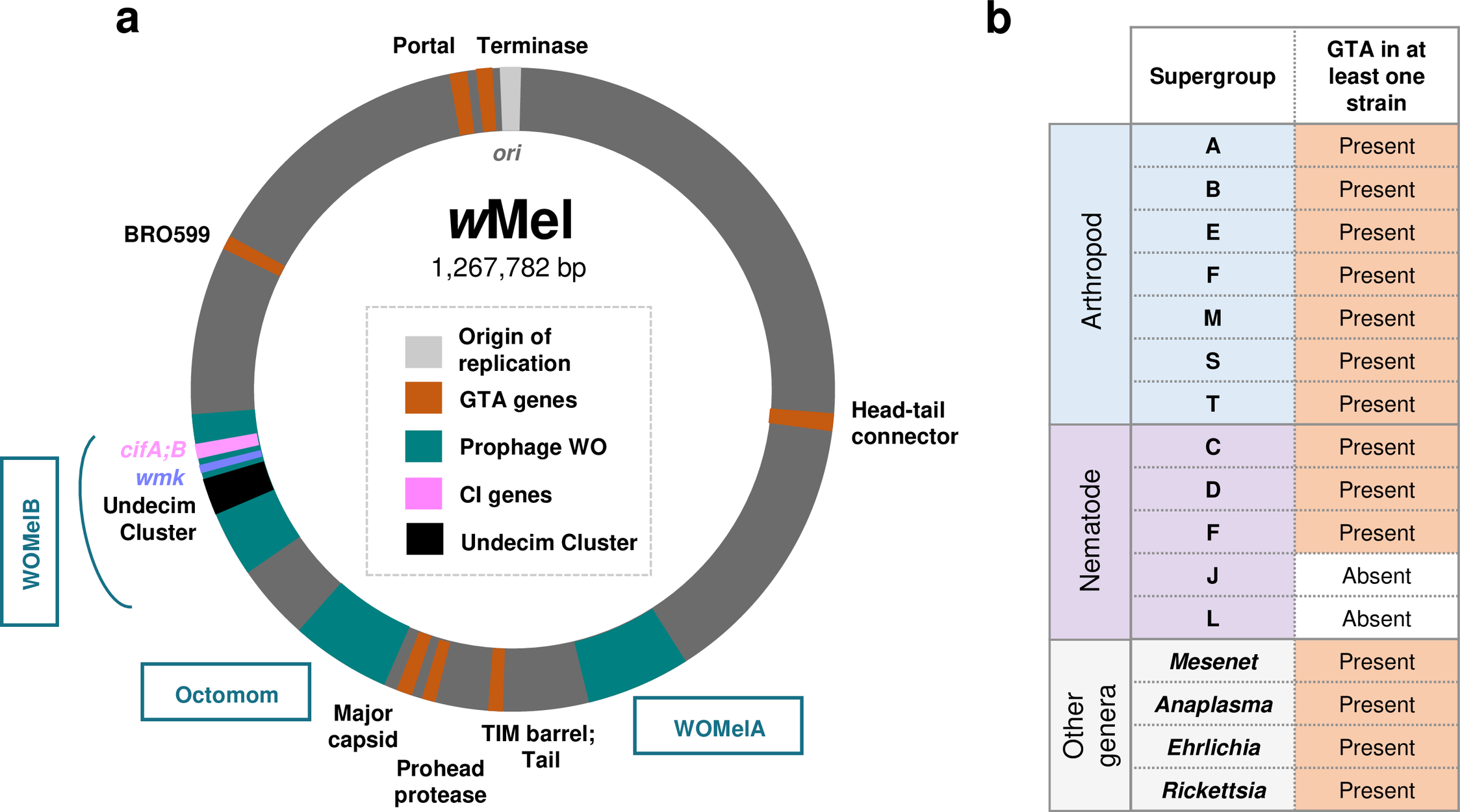

Supplement: S13 Fig — (a) Circular wMel contains three prophage WO-like regions (teal) and multiple genes with homology to GTAs (orange) scattered throughout the genome, illustrated relative to the putative origin of replication (ori, gray). The Undecim Cluster is highlighted in black, cifA;B are highlighted in pink, and wmk is highlighted in purple. (b) GTAs are present in at least one strain of each Wolbachia Supergroup except Supergroups J and L. They are also present in closely related Anaplasmataceae genera. (TIF) [file pgen.1010227.s013.tif]

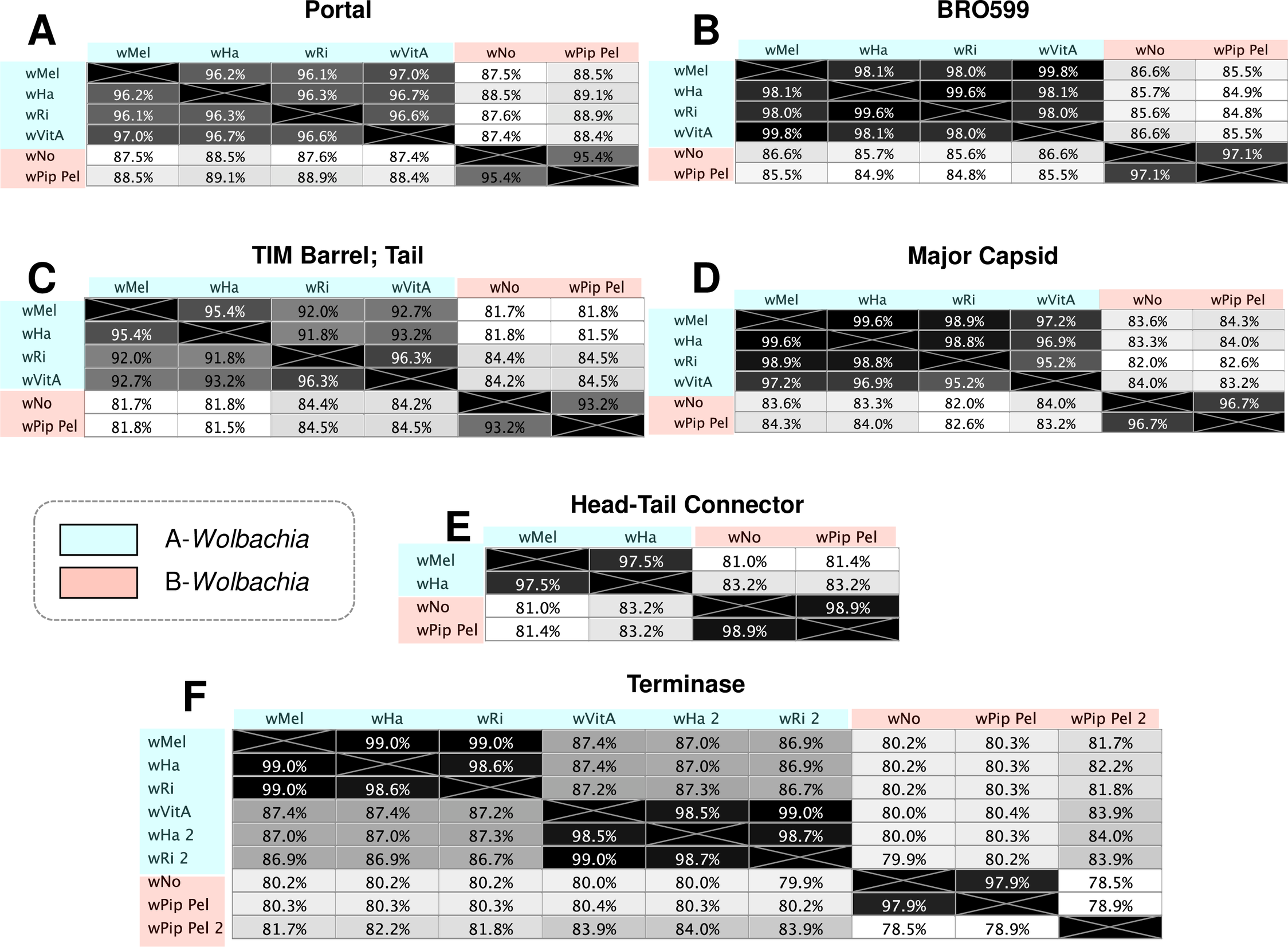

Supplement: S14 Fig — Nucleotide alignments of GTA genes (a) portal, (b) BRO599, (c) TIM barrel, (d) major capsid, (e) head-tail connector, and (f) terminase indicate strict delineation based on Wolbachia supergroup. This supports evolution with the Wolbachia chromosome rather than independent evolution of a phage genome. (TIF) [file pgen.1010227.s014.tif]
